# Surgical Treatment of Fibrous dysplasia in the Maxillary Bone of a 12 Year-Old Girl: A Case Report

**DOI:** 10.29252/wjps.10.3.126

**Published:** 2021-09

**Authors:** Sahand Samieirad, Mohammad Mehdi Momtaz, Nooshin Mohtasham, Farzaneh Mohammadzadeh, Niloofar Ebrahimzadeh, Elahe Tohidi

**Affiliations:** 1 Oral and Maxillofacial Diseases Research Center, Mashhad University of medical sciences, Mashhad, Iran.; 2 Student Research Committee, Faculty of Dentistry, Mashhad University of Medical Science, Mashhad, Iran.; 3 Student Research Committee, Faculty of Dentistry, Zahedan University of Medical Science, Zahedan, Iran.

**Keywords:** Fibrous dysplasia, Maxilla, Computed tomography, Female

## Abstract

Fibrous dysplasia is a rare bony disorder with recurrent character distinguished by abnormal fibro-osseous tissue. One or more bones may be involved in this lesion; however, the maxilla is the most commonly affected bone in the maxillofacial region. Here, we present an interesting case of a surgically treated 12-year-old adult female patient with a diagnosis of craniofacial fibrous dysplasia (CFD) in the maxillary bone with an invasive expansion to the orbital bone who was referred to the Department of Oral and Maxillofacial, Mashhad Dental School, Mashhad, Iran, in Apr 2021. The patient was treated under general anesthesia by the surgical recontouring and bone shaving of the tumor. Several factors should be considered in choosing the best treatment such as pathological features of the lesion, patient’s age, and risk of recurrence. However, surgical treatment, in this case, was beneficial to help the patient resuming a normal life. There is no published report describing bone recontouring and shaving management in an invasive case of craniomaxillofacial FD before the age of skeletal maturity due to the psychological effect of the deformity.

## INTRODUCTION

Fibrous dysplasia (FD) is described as a sporadic, benign congenital disorder of bone development characterized by an abnormal mixture of fibrous and osseous elements replacing cancellous bone^1^^-^^3^. It is the most frequent condition among the Fibro Osseous Lesions (FOL) and it comprises 2.5% of all bone tumors^4^^,^^5^.

The etiology of this entity is still unrevealed but somatic post zygomatic mutations in the gene GNAS in a subunit of the stimulatory G protein could be the cause of it^6^^-^^8^. FD is categorized to monostotic or polyostotic type, clinically. The monostotic type is a single, isolated lesion. Nevertheless, the polyostotic type may affect several bones simultaneously^9^^, ^^10^. The monostotic type of FD is found in 70% of cases and has no gender predisposition and is more frequent in young population with the typical onset in the age of ten, and is usually diagnosed with painless swelling^2^^, ^^8^^, ^^11^^, ^^12^. Polyostotic form is commonly seen in the females and has shown a predilection on one side of the body^7^^, ^^8^. This form is the chief cause of craniofacial fibrous dysplasia (CFD) involvements that mainly occurs in maxilla bone^13^^, ^^14^. The affected area is weakened, making it vulnerable to fractures and deformities, which causes pain and functional difficulties^10^^, ^^15^. The involvement of at least two bones coexisting with brown-to-white pigmentation characterizes Jaffe-Lichtenstein Syndrome^8^. It also can be detected, in the case of McCune–Albright Syndrome (MAS), in combination with hyperfunctioning endocrinopathies and brown-to-brown pigmentation on the skin^8^^, ^^16^.

The clinical signs are higher volume of slow growth with bulging of the involved region and, in great proportions, facial asymmetry^8^^, ^^17^. Major symptoms could be a rapid bone expansion, sinusitis, nasal obstruction, and visual loss, as well as pain and paresthesia^5^^, ^^8^^, ^^10^.

The diagnosis is based on history, clinical examinations, histopathological and the radiographic examinations^5^^,^^10^^,^^14^. Radiographically, the characteristic “ground glass” appearance, of mixed radiolucency/opacity, may be seen resulting from the defective mineralization of immature dysplastic bone^16^^,^^18^. Histologically, curvilinear trabeculae of woven bone, with no osteoblastic rimming, entangled within a bland fibrous stroma without any cellular features of malignancy can be detected^9^^, ^^19^.

To manage the disease, three general approaches are monitoring, medical management, or surgery depending on the location of the lesions, age of the patient, and the patient’s views as well as serial clinical examinations^2^^, ^^5^. The surgical treatment of CFD may be postponed until the age of reaching skeletal maturity, when the expansion of fibrous dysplasia ceases^14^. However, in cases of functional or aesthetic impairments, resection or osteoplasty with cosmetic recontouring performed with curettage, bone grafting, and rarely internal fixation is recommended^2^^, ^^8^^, ^^20^.

There is no published report describing bone recontouring and shaving management in an invasive case of craniomaxillofacial FD before the age of skeletal maturity due to the psychological effect of the deformity. Thus, we aimed to demonstrate the practicality of surgery in bringing a 12-year-old girl patient back to her normal life and the clinical, imaging, laboratory, histological, and development of the case aspects will be discussed.

## CASE REPORT

The study was approved by the local Ethics Committee under the code: IR.MUMS.REC.1400.148. Informed consent was taken from the patient. 

**Fig. 1:**
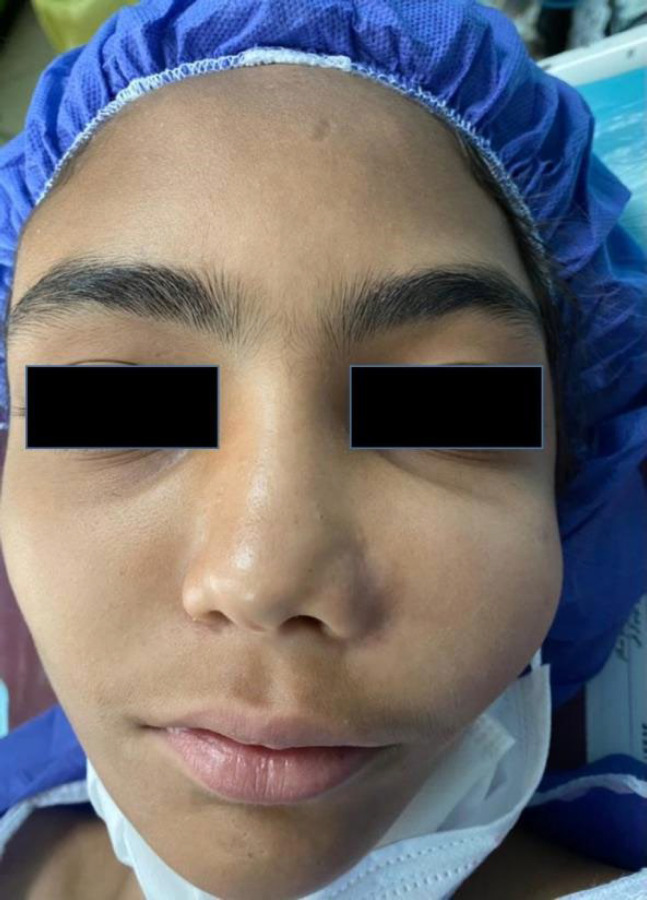
The extraoral clinical view of the patient

A 12-year-old adult girl was referred to the Department of Oral and Maxillofacial, Mashhad Dental School, Mashhad, Iran, in Apr 2021 complaining of a swelling in the left zygomatic-maxillary region started since 2 yr ago and wanted to improve her facial appearance. In her past medical history, the patient reported no systemic diseases as well as no disease-associated syndromes like Jaffe-Lichtenstein, or McCune–Albright Syndrome. Moreover, her hematologic test revealed no abnormal changes. At the extra-oral clinical examination, facial asymmetry was observed in an inferior-superior view, presenting a hard swelling in the left side of the face. There was no lymphadenopathy, skin pigmentation, or any other alteration of skin color (Figure 1).

**Fig. 2:**
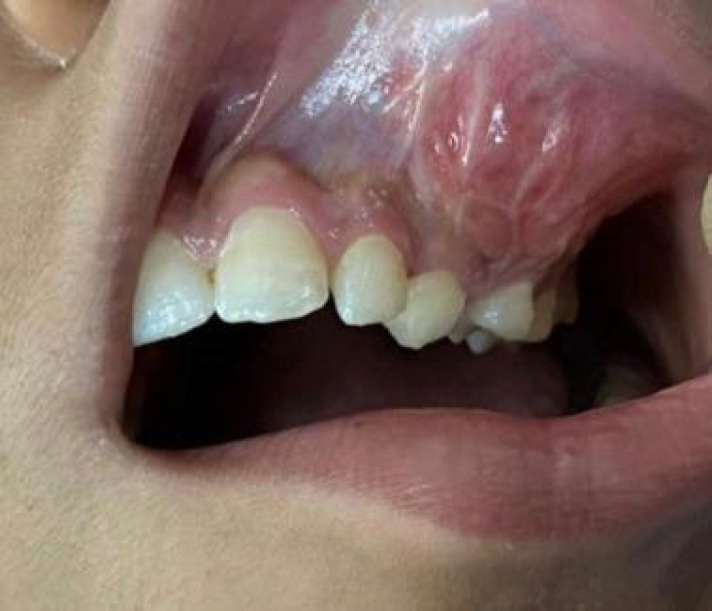
The intraoral clinical examination of the patient

On the intraoral examination, there was a painless mass in the left maxillary region from the canine to the maxillary tuberosity. The oral mucosa had a normal appearance, without any ulcerations. Furthermore, a hard consistency was observed during the clinical palpation of the lesion (Figure 2).

**Fig. 3:**
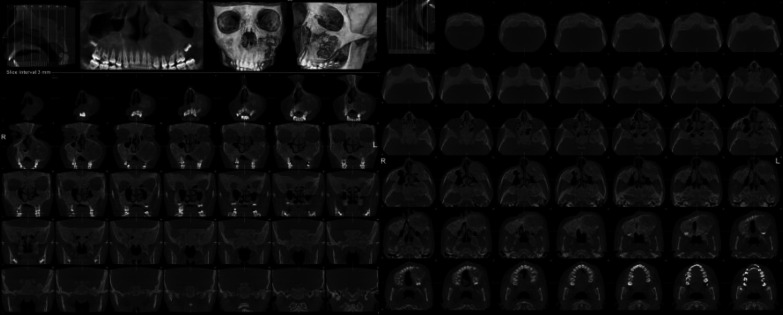
Computed tomography scan of the patient

Computed tomography (CT) scan revealed an expansive left maxillary mass with pathological change in trabecular pattern in the skull base area as a form of the multicentric and bilateral lesion. Involvement of skull foramina and left maxilla palate was observed along with compression of the left nasal cavity. Face CT in coronal and axial sections, revealed a hyper-dense glass density of an insufflating lesion in the maxillary bone expanded vertically to the inferior rim of the orbital bone. It has also partially occupied the left maxillary sinus and the ipsilateral nasal cavity and lowered the hard palate with involvement of the dental alveoli. Mentioned characteristics proposed a possibility of congenital accompanying with the aneurysmal bone cyst or giant cell reparative granuloma (Figure 3). Based on the clinical assessment, CT examinations and incisional biopsy a diagnosis of craniofacial FD was deduced.

***Surgical approach***

All procedures performed in this study involving the human participant were following the ethical standards of our institutional Ethics Committee, Mashhad University of Medical Sciences, Mashhad, Iran, and in accordance with the 1964 Helsinki declaration. 

Due to the recurrence possibility of disease, cases of CFD who are under the age of full skeletal maturity known to be between 16 to 17 yr old in females, are usually told to wait until reaching it. However, because the lesion had created an unfavorable shape in the patient’s face and the psychological impact of it, the surgical treatment was performed sooner. 

**Fig. 4:**
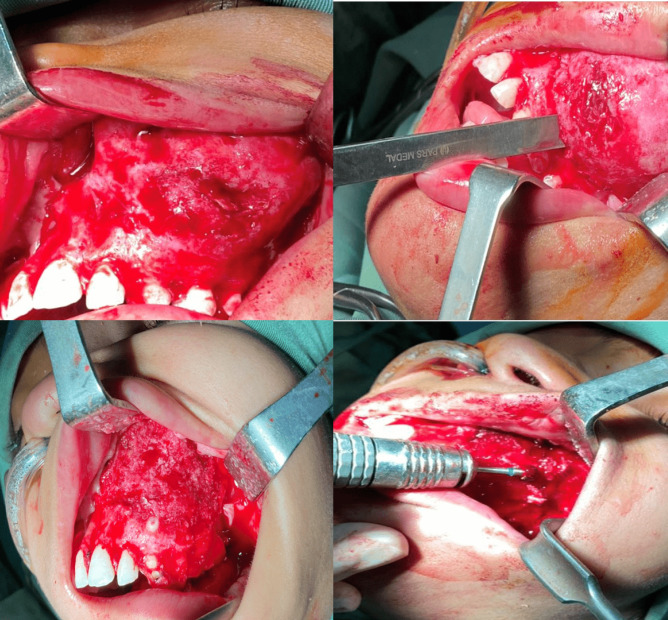
Surgical recontouring and bone shaving of the left maxillary mass

After obtaining the informed consent from the patient, the surgeon decided to perform the surgical recontouring and bone shaving under general anesthesia. Afterward, a vestibular incision was made in the upper left vestibule fundus to access the lesion, then a mucosal flap was removed and to access the affected bone. With performing osteotomy, we removed the extensive woven mass of the bone. Eventually, shaving with a large round burr was carried out (Figure 4). 

**Fig. 5:**
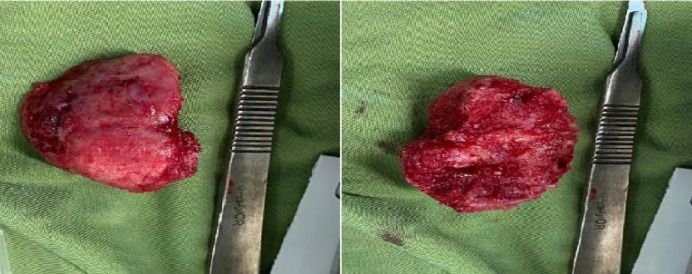
Clinical view of the main resected tumor

Macroscopically, a removed creamy-brown fragment of fibro bony tissue and a spongy bone lesion in dimensions of 4.5×3.5×3 cm can be seen. Furthermore, the serial cut sections revealed homogenous creamy-whitish surfaces (Figure 5).

**Fig. 6:**
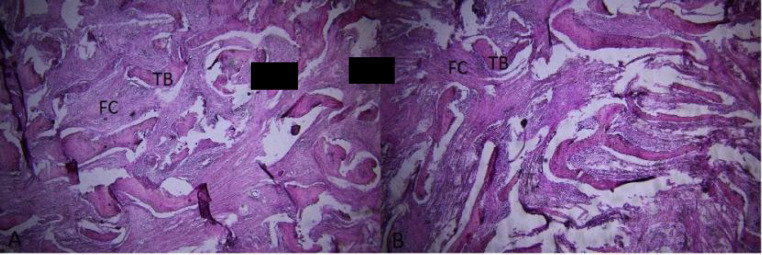
Histopathologic test shows a stroma within irregular shaped trabeculae of woven bone. (H&Estain, ×100) Light Microscope (Lambred, American) Preparing a photograph of the desired areas in the slides by a HP microscope equipped with a camera. (Canon, 650D)

**Fig. 7:**
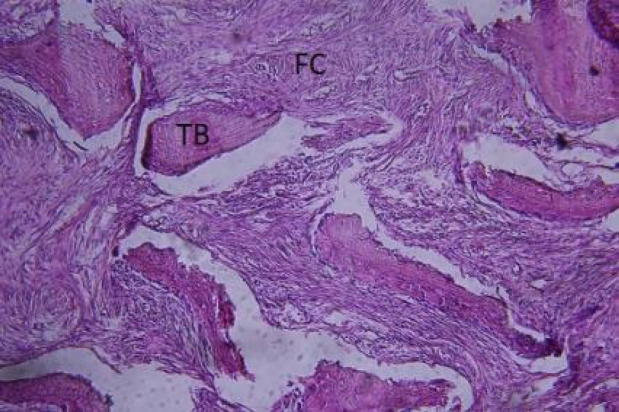
Histopathologic exam shows a fibrocellular stroma within irregular shaped woven bone. (H&Estain, ×400)

The histopathological examination of the surgically removed sections from the mass showed immature woven bone composed of thin and irregularly cancellous trabeculae with devoid of osteoblastic rim surrounded by fibroblastic stroma composed of benign spindle cells arranged in a storiform pattern with scattered laminated bodies of calcification. No dysplasia metaplasia is seen. No mitosis or necrosis are seen. These histopathological findings confirmed the clinical diagnosis of fibrous dysplasia, cementomatous variant known as fibrous cementoma (Figure 6 and 7).

**Fig. 8:**
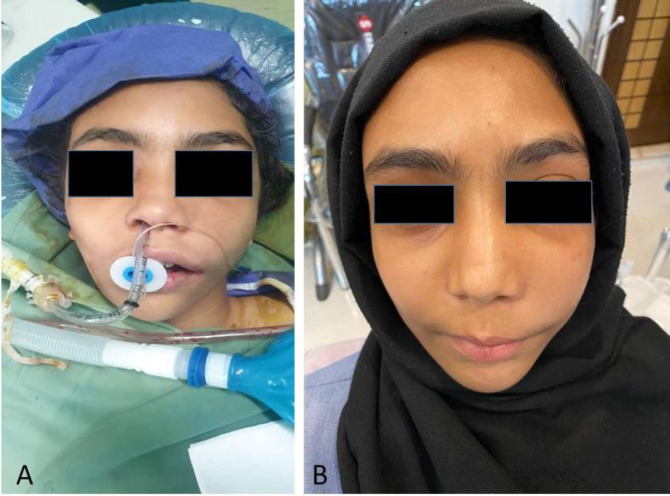
Postoperative photograph: (A) right after the operation, (B) 6 month later

After surgical treatment, the patient experienced satisfactory aesthetics, enhanced symmetry and absence of scarring. A six-month postoperative follow-up of the patient was showed no problem, infection, and recurrence (Figure 8).

## DISCUSSION

Fibrous dysplasia is an unusual skeletal abnormality and an arrested maturation at the woven bone stage^3^^, ^^14^^, ^^21^. According to the recent literature, it is due to a defective mesenchymal tissue development and does not have a relation with hereditary factors^8^^, ^^22^. It is more frequent in females, usually its slow growth stabilizes at puberty, 37% of the cases are recurrent and remain asymptomatic up to 10 yr of age^2^^, ^^8^. Classifications of disease are monostotic, polystotic, and MAS^1^^, ^^9^^, ^^10^^, ^^14^. The polyostotic form can be divided into three subtypes: Craniofacial, which only involves craniofacial bones mostly maxilla; Jaffe-Lichtenstein and Albright syndrome with prominence for early puberty in girls^8^^, ^^16^^, ^^23^.

Regarding the location of the craniofacial FD lesion(s), the symptoms and signs may differ^8^^, ^^9^. The progression of the lesion may cause aesthetic impairment and deformities such as facial asymmetry, sinusitis, orbital dystopia, nasal malfunction^14^^, ^^23^. Clinical manifestations of this case were also found by several authors^1^^,^^8^^,^^10^^,^^22^, such as volume increase in the region, hard consistency on palpation, functional impairment, facial asymmetry. In the report, the patient only complained about expansive facial swelling in left the zygomatic-maxillary region with partial involvement of orbital bone.

CT is the test of choice for the study of lesion(s), evaluation of its extension, and surgical preparation^8^^, ^^24^. There are three general radiographic patterns of CFD including: Ground glass appearance with mixed radiodense and radiotransparent areas; sclerotic, and cystic patterns. In the case reported, tomographic images revealed changes in the pattern of osseous trabeculae with a radiopaque view in the skull base area in the form of multicentric and bilateral lesions. Moreover, the evidence of compromising the left maxillary and zygomatic areas of the face, partial involvement of orbital bone, skull foramina as well as the region of nasal cavity was discovered. It is essential for maxillofacial surgeons to use the CT scans before the surgical procedure to evaluate the actual size of the lesion, anatomical structures, and their involvements. Furthermore, this modality assists the surgeons to estimate the extent of surgical ostectomy and osteoplasty^8^.

The differential diagnosis includes benign lesions: ossifying fibroma, eosinophilic granuloma, Paget’s disease, osteochondroma, giant cell reparative granuloma, aneurysmal bone cyst; and malignancies like metastatic osteoblastic lesions^3^^, ^^8^^,^^ 9^. The initial diagnosis based on clinical aspects along with imaging findings was congenital fibrous dysplasia but the histopathological examination was also performed to confirm it.

Currently, there are no uniformly accepted guidelines for the treatment of CFD, but the age of the patient and the location, size, and biological behavior of the lesion should be considered^9, 14^. Possible remedies could be partial resection, bone remodeling, surgical recontouring, and bone shaving/debulking to achieve cosmetic benefits^1^^, ^^8^^, ^^23^. Surgical treatment is recommended whenever clinical symptoms occur and to relieve intractable pain and skeletal deformity^14^^, ^^22^. Nevertheless, it is not suggested to perform before the age of 18 yr due to the possibility of recurrence because bone growth is still active^2^^, ^^25^. The patient of the exposed clinical case had an important impairment of the left zygomatic-maxillary region. Due to the psychological effect of extensive facial deformity and, with aesthetic purpose, the surgical recontouring and bone shaving was proposed to be performed.

Histopathologic examination shows misshapen bony trabeculae surrounded by loose fibrous tissue with scattered hematopoeitic foci and destruction of the affected bone agreeing with the authors^2^^, ^^8^^, ^^14^^, ^^22^.

A specimen of the lesion was sent for histopathological testing for microscopic examination and it was compatible with diagnosis of craniofacial fibrous dysplasia.

In patients with CFD, regarding the moderate rate of recurrence of the lesion(s), clinical and radiological follow-up by CT is fundamental and it may reach the rate of 37% according to some articles ^8^.

FC: fibrocellular tissue TB: trabeculae of bone

## CONCLUSION

When it comes to the clinical case, FD is regarded as a disorder that might cause functional and aesthetic impairment. When deciding whether or not to treat fibrous dysplasia, the patient’s age, the existence or absence of facial asymmetry/involvement and future rehabilitation should all be taken into account.

Because it is a relapsing tumor, it is critical to remove as much tissue as possible without inflicting mutilation, functional impairments, or lesions of noble structures. The surgical procedure in fibrous dysplasia, a restricted contouring or radical osteoplastic flap operation is used to enhance facial looks and, in certain cases, functional problems. The follow-up is of fundamental importance in order to detect relapses.

## Funding

This study was self-funded.

## CONFLICT OF INTEREST

The authors declare that there is no conflict of interests.

## References

[1] Ahmad M, Gaalaas L (2018). Fibro-osseous and other lesions of bone in the jaws. Radiologic Clinics.

[2] Rahman A, Madge S, Billing K (2009). Craniofacial fibrous dysplasia: clinical characteristics and long-term outcomes. Eye.

[3] Hong I, Kang DC, Leem D-H, Baek J-A, Ko S-O (2020). An unusual presentation of non-specific cystic degeneration of craniofacial fibrous dysplasia: a case report and review of literature. Maxillofac Plast Reconstr Surg.

[4] Fan Y, Liu J, Zhang C, Zhang Z, Yang H, Hu J (2017). Maxillofacial fibrous dysplasia: A clinical analysis of 72 cases. Biomed Res.

[5] Ricalde P, Horswell BBJJoo, surgery m (2001). Craniofacial fibrous dysplasia of the fronto-orbital region: a case series and literature review. J Oral Maxillofac Surg.

[6] Alkhaibary A, Alassiri AH, Alsalman M, Edrees SJJorcr (2019). Unusual presentation of fibrous dysplasia in an elderly patient. J Radiol Case Rep.

[7] Yang H-Y, Su B-C, Hwang M-J, Lee Y-P (2018). Fibrous dysplasia of the anterior mandible: A rare case report. Tzu-Chi Medical Journal.

[8] Pinto MD, Braz G, Santos RG, Polido D, Paixão JR, de Melo SH (2018). Fibrous Dysplasia in Maxillary Bone: Case Report. Int J Oral Dent Health.

[9] DiCaprio MR, Enneking WFJJ (2005). Fibrous dysplasia: pathophysiology, evaluation, and treatment. JBJS.

[10] Lee J, FitzGibbon E, Chen Y (2012 ). Clinical guidelines for the management of craniofacial fibrous dysplasia. In Orphanet J Rare Dis.

[11] Ebenezer DV (2020). Maxillary fibrous dysplasia–a case report. European Journal of Molecular & Clinical Medicine.

[12] Dorfman HDJIjosp (2010). New knowledge of fibro-osseous lesions of bone. Int J Surg Pathol.

[13] Yang L, Wu H, Lu J, Teng LJJocs (2017). Prevalence of different forms and involved bones of craniofacial fibrous dysplasia. J Craniofac Surg.

[14] Assaf AT, Benecke AW, Riecke B (2012). Craniofacial fibrous dysplasia (CFD) of the maxilla in an 11-year old boy: A case report. J Craniomaxillofac Surg.

[15] Hwang D, Jeon J, Hong SH, Yoo HJ, Choi J-Y, Chae HD (2020). Radiographic follow-up of fibrous dysplasia in 138 patients. AJR Am J Roentgenol..

[16] Burke A, Collins MT, Boyce AMJOd (2017). Fibrous dysplasia of bone: craniofacial and dental implications. Oral Dis.

[17] Sentürk M, Külahli I, Emiroğlu A (2003). [Fibrous dysplasia in the head and neck region: a report of three cases]. Kulak Burun Bogaz Ihtis Derg.

[18] Lietman SA, Levine MAJPER (2013). Fibrous dysplasia. Int J Surg Pathol.

[19] Riddle ND, Bui MMJAop, medicine l (2013). Fibrous dysplasia. Int J Surg Pathol.

[20] Boyce AM, Chong WH, Yao J (2012). Denosumab treatment for fibrous dysplasia. GeneReviews.

[21] Yasuoka T, Takagi N, Hatakeyama D, Yokoyama KJOo (2003). Fibrous dysplasia in the maxilla: possible mechanism of bone remodeling by calcitonin treatment. Oral oncology.

[22] Godse A, Shrotriya S, Vaid NJJops (2009). Fibrous dysplasia of the maxilla. J Pediatr Surg.

[23] Bowers CA, Taussky P, Couldwell WTJNr (2014). Surgical treatment of craniofacial fibrous dysplasia in adults. Neurosurgical Review.

[24] Fuster A, Rodríguez-Pereira C, JM GNJAoe (2002). Monostotic fibrous dysplasia of the frontal sinus with orbital extension. Neurosurgical Review.

[25] Mendonça Caridad JJ, Platas F Jr (2008). Fibrous dysplasia of the mandible: Surgical treatment with platelet-rich plasma and a corticocancellous iliac crest graft-report of a case. Oral Surg Oral Med Oral Pathol Oral Radiol Endod.

